# Island-specific evolution of a sex-primed autosome in a sexual planarian

**DOI:** 10.1038/s41586-022-04757-3

**Published:** 2022-06-01

**Authors:** Longhua Guo, Joshua S. Bloom, Daniel Dols-Serrate, James Boocock, Eyal Ben-David, Olga T. Schubert, Kaiya Kozuma, Katarina Ho, Emily Warda, Clarice Chui, Yubao Wei, Daniel Leighton, Tzitziki Lemus Vergara, Marta Riutort, Alejandro Sánchez Alvarado, Leonid Kruglyak

**Affiliations:** 1 Department of Human Genetics and Department of Biological Chemistry, University of California, Los Angeles, Los Angeles, CA USA; 2 Howard Hughes Medical Institute, Chevy Chase, MD USA; 3 Departament de Genètica, Microbiologia i Estadística, Institut de Recerca de la Biodiversitat, Universitat de Barcelona, Barcelona, Spain; 4 Department of Biochemistry and Molecular Biology, Institute for Medical Research Israel-Canada, Hebrew University of Jerusalem–Hadassah Medical School, Jerusalem, Israel; 5 Institute of Reproductive Medicine, Henan Provincial People’s Hospital, Zhengzhou University, Zhengzhou, China; 6 Stowers Institute for Medical Research, Kansas City, MO USA

**Keywords:** Genome evolution, Haplotypes, Evolutionary genetics, Chromosomes, Genome

## Abstract

The sexual strain of the planarian *Schmidtea mediterranea*, indigenous to Tunisia and several Mediterranean islands, is a hermaphrodite^1,2^. Here we isolate individual chromosomes and use sequencing, Hi-C^3,4^ and linkage mapping to assemble a chromosome-scale genome reference. The linkage map reveals an extremely low rate of recombination on chromosome 1. We confirm suppression of recombination on chromosome 1 by genotyping individual sperm cells and oocytes. We show that previously identified genomic regions that maintain heterozygosity even after prolonged inbreeding make up essentially all of chromosome 1. Genome sequencing of individuals isolated in the wild indicates that this phenomenon has evolved specifically in populations from Sardinia and Corsica. We find that most known master regulators^5–13^ of the reproductive system are located on chromosome 1. We used RNA interference^14,15^ to knock down a gene with haplotype-biased expression, which led to the formation of a more pronounced female mating organ. On the basis of these observations, we propose that chromosome 1 is a sex-primed autosome primed for evolution into a sex chromosome.

## Abstract (web summary)

Assembly and analysis of the *Schmidtea mediterranea* genome indicate that this planarian’s chromosome 1 may be evolving into a sex chromosome.

## Main

Sex chromosomes evolve from homologous autosomes that acquire sex-determining genes and lose their ability to recombine^16–21^. As such, sex chromosome evolution and recombination suppression are closely associated^16–21^. However, because direct evidence of such homologous autosomes primed for evolution into sex chromosomes is difficult to capture, little is known about the molecular signatures associated with the evolution of recombination suppression.

The freshwater planarian *Schmidtea mediterranea*, an important model organism for studies of regeneration^22,23^, exists as asexual and sexual reproductive strains. The sexual strain is distributed mostly in Tunisia and on the islands of Sardinia, Corsica and Sicily^1^. The sexual strain is a simultaneous hermaphrodite that develops both male and female reproductive systems in the same adult individual and obligately outcrosses to fertilize other individuals^24,25^. Individuals in the asexual strain do not develop sexual reproductive systems. We considered that studying chromosome evolution in a simultaneous hermaphrodite might provide insight into the early evolution of a primitive sex chromosome.

*S*. *mediterranea* has four pairs of chromosomes, which are stably diploid. The genome is reported to comprise 774 Mb assembled as 481 non-contiguous series of genomic sequences, or scaffolds^26–28^. A previous study found that approximately 300 Mb of the genome remained heterozygous even after extensive inbreeding of laboratory strains, and that this phenomenon also occurs naturally in wild populations in Sardinia^24^. The two sets of heterozygous alleles were collectively named J and V haplotypes. To define the chromosomal locations of these alleles and investigate the reasons underpinning the persistence of heterozygosity in *S*. *mediterranea*, a detailed assembly of all four chromosomes is needed.

## Chromosome-scale genome assembly

**Fig. 1:**
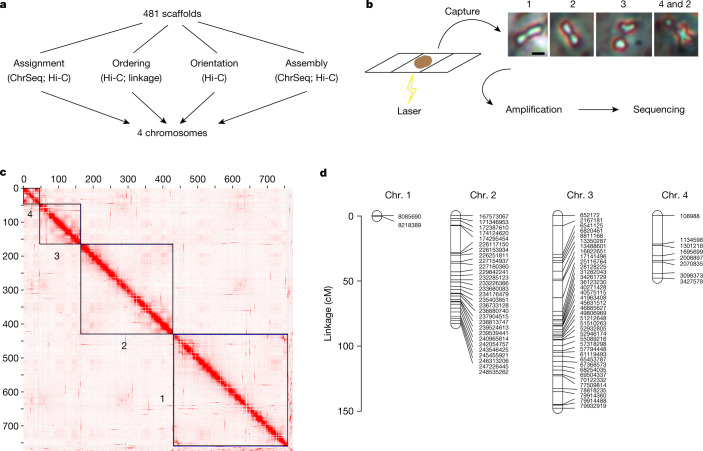
Chromosome-scale genome assembly. **a**, Schematic of the use of Hi-C, ChrSeq and a linkage map to transform 481 scaffolds^26^ into a chromosome-scale genome assembly. **b**, Chromosomes from mitotic cells spread onto membrane slides for laser capture and sequencing. Numbers denote the identity of chromosomes present in the representative samples. **c**, Contact heat map of chromatin interactions indicated by Juicebox^4^; black boxes denote the four chromosomes in the final assembly. **d**, Linkage map generated through LinkageMapView^68^, showing a lack of recombination on chromosome 1. Tick marks and labels indicate genetic markers.

**Extended Data Fig. 1:**
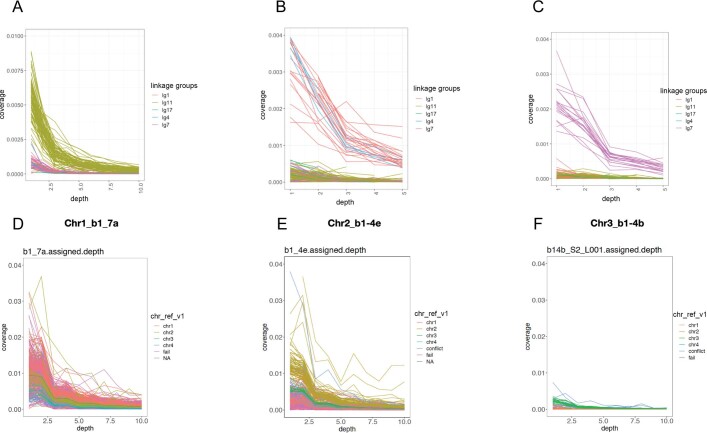
Chromosome assignment by ChrSeq. (A-C) Chromosome sequencing depth of different scaffolds is consistent with linkage group assignment. Linkage groups: lg11 = chr1, lg1+lg4 = chr2, lg7 = chr3, lg17 = chr4. (D-F) Chromosome sequencing depth of different scaffolds is consistent with chromosome assignment in the genome assembly smed_chr_ref_v1. Chromosome sequencing samples: chromosome 1 (A, D); chromosome 2 (B, E); chromosome 3 (C, F).

To transform the 481 scaffolds^26^ into a chromosome-scale genome reference, we carried out chromosome sequencing (ChrSeq)^29,30^ of a laboratory strain, as well as chromatin proximity sequencing by Hi-C^3^ (Fig. 1a). To do so, we dissected individual chromosomes from mitotic cells using laser capture and amplified and sequenced each chromosome individually (Fig. 1b). We examined the sequencing depth of each scaffold across multiple samples of the same and different chromosomes to ensure reproducibility and specificity, respectively (Extended Data Fig. 1). Overall, we successfully amplified and confidently assigned 740 Mb of the 774-Mb genome to one of four chromosomes (Supplementary Table 1).

**Extended Data Fig. 2:**
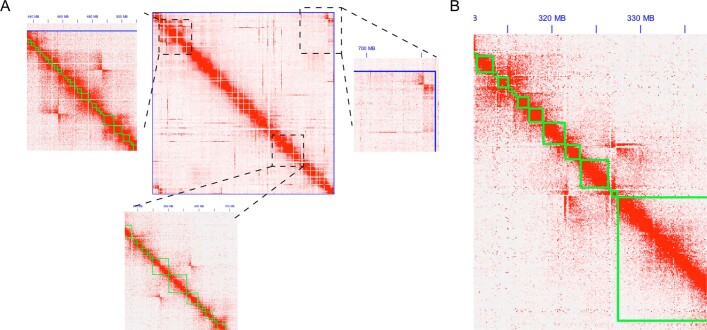
Putative inversions in the planarian genome detected by chromatin contact heatmap. (A) chromosome 1 chromatin contact heatmap showing 3 potential inversions (dashed-line rectangles) visualized in Juicebox. (B) A complex structural variant on chromosome 2. Green outline: dd_Smes_g4 scaffolds. Blue outline: chromosome boundaries.

We used ChrSeq information and chromatin interaction data generated by Hi-C to correct and connect the individual scaffolds within a chromosome into a chromosome-scale genome, hereafter referred to as Smed_chr_ref_v1. The Hi-C data analysed using the SALSA scaffolding algorithm^3^ resolved the 481 scaffolds into 57 super-scaffolds and 104 singletons. The ChrSeq data indicated that 3 of the 57 super-scaffolds were disjointed inter-chromosomal fragments, consistent with the Hi-C contact heat map (Fig. 1c). We split the disjointed super-scaffolds, merged the scaffolds into chromosomes, and ordered and oriented all scaffolds within the chromosomes using Juicebox visualization software^4^ (Methods). Chromosome assignments by ChrSeq alone (Supplementary Table 1) and by Hi-C alone were inconsistent for only 3 of 384 (0.8%) scaffolds (Supplementary Table 2). We manually assigned these three scaffolds to chromosomes according to the Hi-C data. Hi-C also detected 26 inter-chromosomal or intra-chromosomal assembly errors (Supplementary Table 2) in the previous assembly^26^, 5 of which were confirmed by ChrSeq to be inter-chromosomal disjointed scaffolds (Supplementary Table 2). The final genome assembly, Smed_chr_ref_v1 (Fig. 1c), had four chromosomes with a total size of 764 Mb, which is 98.4% of that reported previously^26^. Of the 1.6% of the previous assembly not contained in Smed_chr_ref_v1 by Hi-C, about half (52.6%) of the scaffolds could not be assigned to a chromosome or were assigned to two chromosomes by ChrSeq, indicating lower assembly quality (Supplementary Table 3). Moreover, 33.0% and 12.4% of the scaffolds had chromatin interaction signals with structurally complex regions of chromosomes 1 and 2, respectively (Extended Data Fig. 2), suggesting that they might be alternative assemblies or repetitive sequences. In our new assembly, Smed_chr_ref_v1, the two ends of chromosome 4 were capped by >1,000 copies of the telomere repeat TTAGGG, indicating high assembly quality.

To validate the linearity of the chromosomes in Smed_chr_ref_v1, we generated a linkage map. We crossed two divergent laboratory strains of *S*. *mediterranea*, S2F10b and D5, to produce an F_2_ population, and we genotyped individual worms with RADseq^31^. Eighty markers that were evenly distributed and genotyped in at least 98% of the F_2_ segregants (91 of 93) were used to establish four linkage groups (Fig. 1d and Supplementary Table 4) representing the four chromosomes. The ordering of the 80 markers in the linkage map is consistent with Smed_chr_ref_v1, independently supporting the quality of our chromosome-scale genome assembly. This highly contiguous and complete genome assembly and linkage map together facilitate further genetic and epigenetic investigations of the functions of the genome in this model planarian.

## Chromosome 1 recombination suppression

**Fig. 2:**
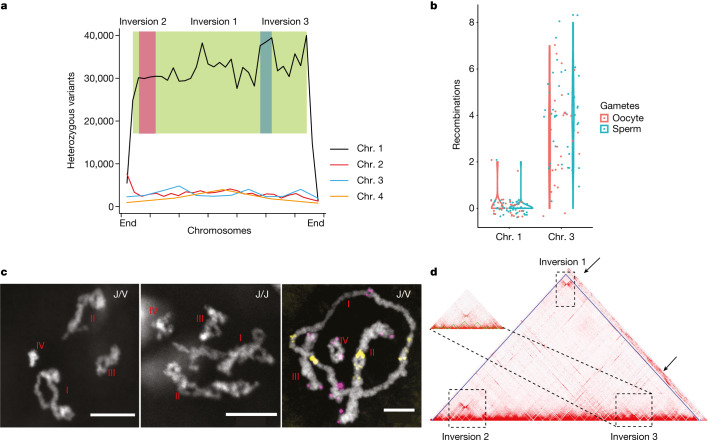
Recombination suppression and inversions on chromosome 1. **a**, Distribution of heterozygous variants maintained in the S2 inbreeding pedigree along the four chromosomes. The *y* axis shows variant counts per 10 Mb. Green, pink and blue boxes represent inversions 1, 2 and 3, respectively. **b**, Number of meiotic recombination events on chromosomes 1 and 3 in oocytes (red) and sperm cells (cyan). Dots represent individual gametes, summarized with violin plots. **c**, Chromosome 1 in late prophase I in oocytes from J/V worms (left and right panels) shows fewer crossovers than in oocytes from J/J worms (middle panel). This experiment was repeated more than 10 times independently with similar results. The right panel shows FISH^42^ results with probes for the telomeric repeat TTAGGG (magenta) and a repeat located near the centromere (yellow). This experiment was repeated more than three times independently with similar results. **d**, Chromatin contact heat map for chromosome 1. Dashed-line rectangles indicate the locations of potential inversions. Arrows indicate chromatin contact signals with inversion regions from unassigned scaffolds.

We next re-examined the heterozygous alleles that define the J and V haplotypes in the newly assembled genome. We found that 87.7% of the genetic markers that remained heterozygous were located on chromosome 1, spanning 333 Mb at a density of 30,148 variants per 10 Mb (Fig. 2a). The remaining 12.3% of the heterozygous markers were located on the other three chromosomes at a density of 3,274 variants per 10 Mb and probably correspond to differences between highly similar copies of repetitive elements rather than true polymorphisms (Supplementary Table 5). All F_2_ worms (*n* = 93) in this study were heterozygous for chromosome 1 but not for chromosomes 2–4 (Supplementary Table 6), which is consistent with the previous study^24^. Hence, we concluded that the J/V haplotypes were on chromosome 1.

Our linkage map revealed an extremely low rate of recombination on chromosome 1 of only 0.5 cM for the entire chromosome (Fig. 1d). This is particularly notable because, at 333 Mb, chromosome 1 was the largest of the four chromosomes, containing more than 40% of the genome.

**Extended Data Fig. 3:**
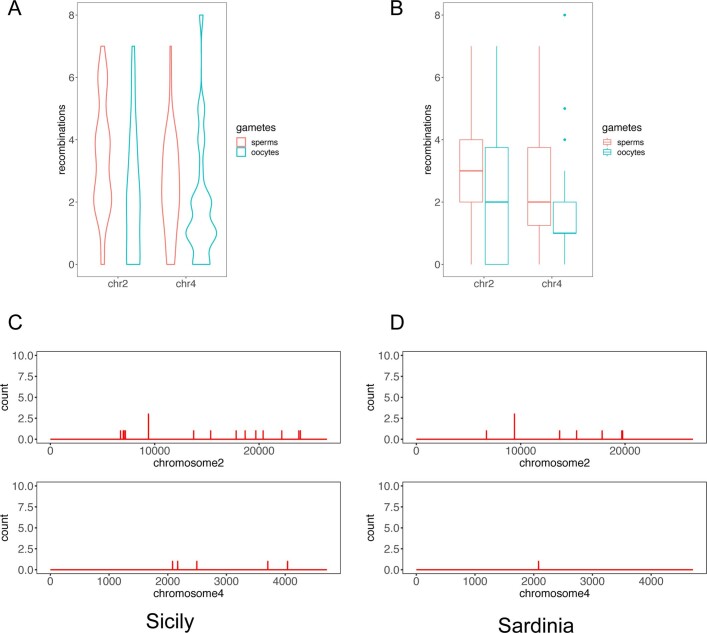
Recombination in gametes and heterozygosity distribution in the wild on chromosomes 2 and 4. (A-B) Number of recombinations in oocytes and sperm on chromosomes 2 and 4 summarized as a violin plot (A) and a box plot (B). The median number of recombinations on chromosome 2 is 3 for sperm and 2 for oocytes, derived from a total of 49 sperm and 38 oocytes. The median number of recombinations on chromosome 4 is 2 for sperm and 1 for oocytes, derived from a total of 50 sperm and 26 oocytes. Chr2 sperm: min = 0, max = 7, median = 3, first quartile = 2, third quartile = 4. Chr2 oocytes: min = 0, max = 7, median = 2, first quartile = 0, third quartile = 4; Chr4 sperm: min = 0, max = 7, median = 2, first quartile = 1, third quartile = 4; Chr4 oocytes: min = 0, max = 3, median = 1, first quartile = 1, third quartile = 2. (C-D) Very few sites are consistently heterozygous in more than 80% of the wild isolates on chromosomes 2 and 4 in Sicily (C) and in Sardinia (D).

To directly examine whether chromosome 1 can recombine, we sequenced 45 single sperm cells and 28 single oocytes from J/V line S2 (Fig. 2d). Gamete sequencing is preferred because recombination events in hatchlings can be selected for by differential fertilization or embryonic lethality. We identified 3,197 single-nucleotide variants (SNVs) on chromosome 1 and 3,312 SNVs on chromosome 3, covering 99% of the length of each chromosome (Supplementary Table 7). The SNVs were distributed at a similar density across 20-Mb windows, with coefficients of variation of 0.38 and 0.31 for chromosomes 1 and 3, respectively (Supplementary Table 7). We observed that 98% of the sperm cells (44 of 45) and 93% of the oocytes (26 of 28) had no crossovers on chromosome 1. By contrast, most sperm cells and oocytes had crossovers on chromosomes 2, 3 and 4 (Fig. 2b, Extended Data Fig. 3a, b and Supplementary Table 7). We thus concluded that recombination on chromosome 1 was strongly suppressed.

**Extended Data Fig. 4:**
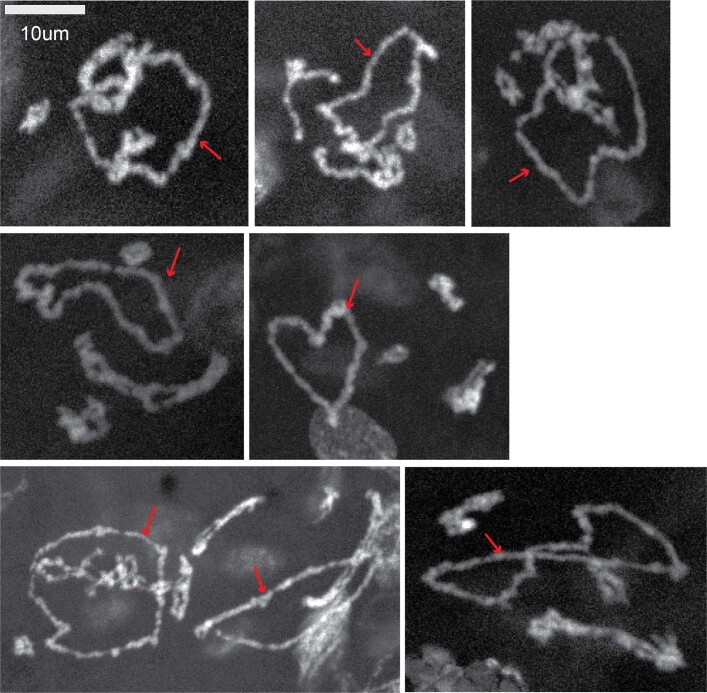
Representative images of late prophase I oocytes with ring-shaped chromosome 1. More than 100 oocytes from > 40 ovaries and > 20 sexual strains were examined for the presence of a ring-shaped chromosome 1. The experiment was repeated >10 times independently with similar results. Chromosome 1 always forms a ring in J/V strains in late prophaseI oocytes. Shown in the figure are chromosomes from 8 different oocytes atlate prophase I. Arrows point to the ring-shaped chromosome 1.

Consistent with this conclusion, we found that during prophase I, when other chromosomes had numerous crossovers, chromosome 1 formed a ring structure in the oocyte in J/V worms but not in J/J worms (Fig. 2c and Extended Data Fig. 4). Fluorescence in situ hybridization (FISH) with telomere probes suggested that crossovers between the two homologous pairs of chromosome 1 occurred only in regions close to the telomeres, leading to the observed ring conformation rather than the side-by-side pairing observed for chromosomes 2, 3 and 4. Furthermore, Hi-C analysis showed that chromosome 1 had three putative inversions, each >20 Mb in size (Fig. 2d); such inversions can cause crossover suppression^32–34^. The rest of the genome had only one large inversion of approximately 10 Mb on chromosome 2 (Extended Data Fig. 2b).

## Island-specific evolution of chromosome 1

**Fig. 3:**
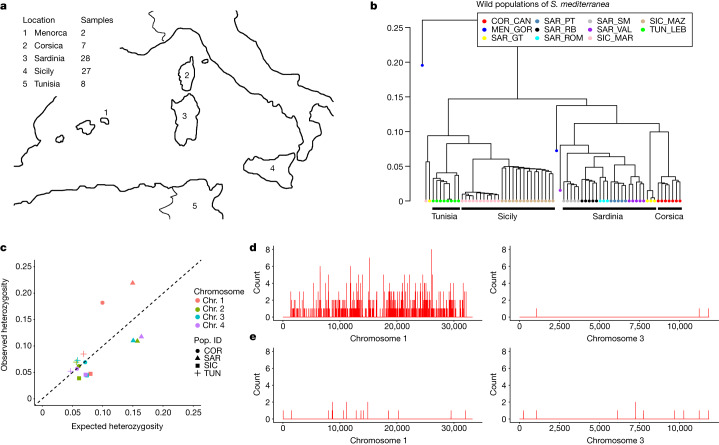
Island-specific evolution of chromosome 1 heterozygosity. **a**, Collection sites of 72 wild isolates from the Mediterranean islands and Tunisia. **b**, Maximum-likelihood tree of 2 asexual isolates from Menorca (MEN_GOR) and 70 sexual isolates from Sardinia (SAR), Corsica (COR), Sicily (SIC) and Tunisia (TUN). Dots denote individual worms, with colours corresponding to their collection sites. **c**, Observed versus expected heterozygosity for each chromosome (denoted by colour) in each population (denoted by shape). The dashed line corresponds to equality between observed values and those expected under Hardy–Weinberg equilibrium; the deviation of the points corresponding to chromosome 1 in the COR and SAR samples from the expectation is notable. **d**, **e**, Number of sites with heterozygosity in >80% of the population per 10-kb window along chromosome 1 (left) and chromosome 3 (right) in Sardinia (**d**) and Sicily (**e**).

**Extended Data Fig. 5:**
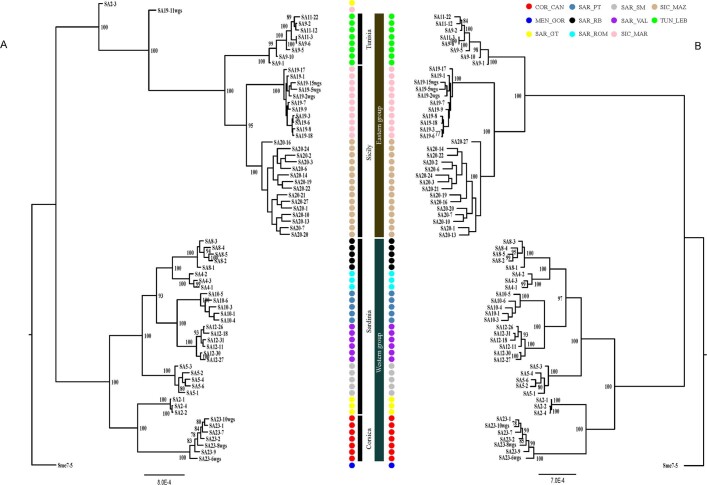
Phylogenetic tree of wild isolates of *Schmidtea mediterranea*. The tree is rooted with one asexual animal from Menorca (Sme7-5). The tree on the left (A) includes two animals from SAR and SIC with higher genome heterozygosity than the cohorts from the same collection sites. Phylogenetic relations agree with the previous designation of eastern and western populations^1^. SAR: Sardinia; COR: Corsica; SIC: Sicily; TUN: Tunisia.

**Extended Data Fig. 6:**
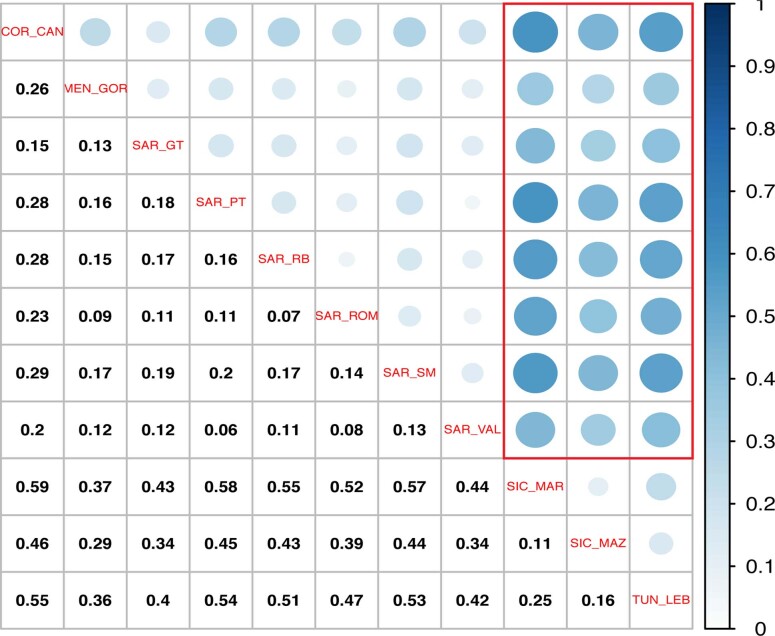
Fixation index (F_ST_) analysis of wild isolates. Pairwise population F_ST_ for all collection sites from Sardinia (SAR), Corsica (COR), Menorca (MEN), Sicily (SIC), and Tunisia (TUN). The size and darkness of the blue circles (top right) correspond to the values of F_ST_ (bottom left).

**Extended Data Fig. 7:**
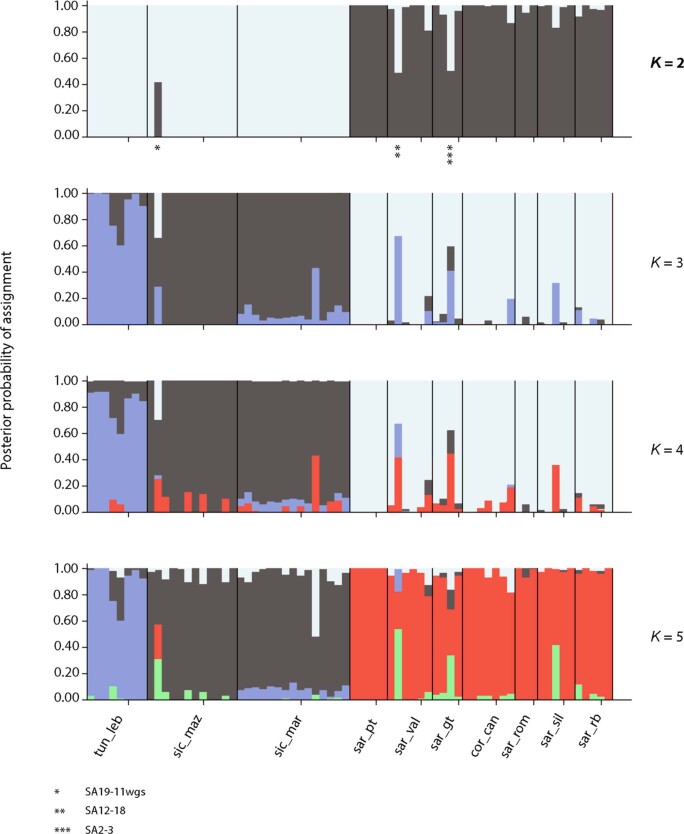
STRUCTURE analysis of wild isolates. Evanno analysis supports a K = 2 split, dividing the samples geographically into Eastern and Western groups. With K = 3, a Tunisian signal was found to be present in all other 3 groups (light blue).

To investigate the genetic diversity of *S*. *mediterranea* in its natural environment and determine whether chromosome 1 J/V heterozygosity occurs throughout the entire species, we used RADseq^31^ to sample the genomes of 70 sexual individuals from Sardinia, Corsica, Sicily and Tunisia and 2 asexual individuals from Menorca^1^ (Fig. 3a). To look for genetic relationships between the individuals, we determined clustering of relatedness measured by identity-by-state pairwise distances^35^ and identified two superclusters. Animals from Sicily were closely related to those from Tunisia, and animals from Sardinia were closely related to those from Corsica (Fig. 3b). Phylogenetic clustering (Extended Data Fig. 5), fixation index (*F*_ST_) values (Extended Data Fig. 6) and analyses with Structure (Extended Data Fig. 7) further supported this observation.

The relatedness of the populations from Sardinia and Corsica suggests that they may share genome characteristics that differ from those of the populations from Sicily and Tunisia. Indeed, animals in the populations from Sardinia and Corsica had greater heterozygosity on chromosome 1 than expected under Hardy–Weinberg equilibrium (Fig. 3c), whereas the heterozygosity on the other three chromosomes in these populations and on all four chromosomes in the other populations closely followed expectations. By analysing the J/V haplotype markers in wild populations, we found that animals from Sardinia and Corsica were heterozygous J/V, whereas those from Sicily and Tunisia were homozygous J/J. Moreover, the animals from Sardinia (*n* = 28) had many sites that were heterozygous in more than 80% of the individuals and were distributed along the length of chromosome 1 except near the ends; conversely, the animals from Sicily (*n* = 27) had very few such heterozygous sites (Fig. 3d, e and Supplementary Table 8). Few such heterozygous sites were observed on chromosomes 2, 3 and 4 in either population (Fig. 3d, e and Extended Data Fig. 3c, d). These analyses suggest that chromosome 1 specifically evolved and diverged on the islands of Sardinia and Corsica.

## A sex-primed chromosome

**Fig. 4:**
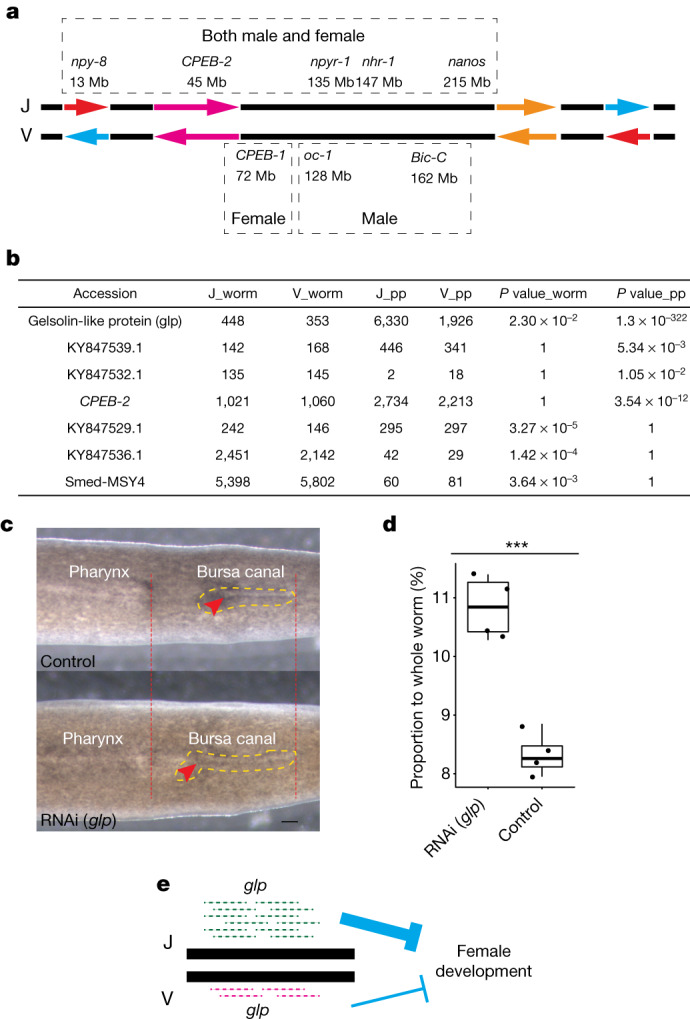
Acquisition and haplotype-specific expression of sex-related genes. **a**, Schematic diagram of the inferred structure of the J and V haplotypes on chromosome 1. Arrows denote putative inversions. Names, positions and sex specificity are shown for the eight genes on chromosome 1 with known key roles in the development of the reproductive system. **b**, Comparison of read counts for the J and V alleles of key genes in the transcriptomes of whole sexually mature worms and penis papillae (PP). Bonferroni-corrected *P* values from a two-sided binomial test of equal expression are shown. **c**, Dorsal view of control (top) and *Smed-glp* knockdown (bottom) sexually mature planarians. Red dashed lines and arrows indicate the posterior and anterior ends of the pharynx and the bursa canal, respectively. Scale bar, 400 um. Yellow dashed lines outline the bursa canal. **d**, Quantification of the length of the bursa canal relative to that of the whole worm. The *y* axis shows the percentage of whole-worm length spanned by the bursa canal as a box plot, with individual data points shown (*n* = 4 each for control and knockdown worms). Statistical significance was assessed with a two-sided, two-sample Student’s *t*-test (***P = 0.00029). RNAi (*glp*): minimum, 10.28; maximum, 11.4; median, 10.845; first quartile, 10.375; third quartile, 11.31. Control: minimum, 7.95; maximum, 8.85; median, 8.26; first quartile, 8.06; third quartile, 8.6. **e**, Model of negative regulation of bursa canal development by *Smed-glp* alleles.

To gain insight into the island-specific suppression of recombination on chromosome 1, we examined the genes located on this chromosome, which contained 39% of all annotated genes. Five of the seven known master regulators of the reproductive system were found on chromosome 1, including *nanos*^5^, *nhr-1* (refs. ^8,9^), *npy-8* (ref. ^6^, *npyr-1* (ref. ^11^) and *CPEB-2* (ref. ^12^) (*ophis*^11^ and *boule-2* (ref. ^10^) were the two exceptions) (Fig. 4a). Knockdown of any one of these master regulators leads to depletion of both male and female reproductive tissues^5,6,8–12^. The presence of these genes with crucial roles in sexual development on chromosome 1 suggests that chromosome 1 integrity is important for the maintenance of sexual reproduction. Indeed, the asexual lineage of *S*. *mediterranea*, which is devoid of any reproductive organs^23^, had a translocation from chromosome 1 to chromosome 3 and probably evolved through loss of function of one or more of these genes.

Chromosome 1 also contained three of the four known master regulators of male or female reproductive tissues (*CPEB-1* (ref. ^12^), *onecut*^13^ and *Bic-C*^5^; *dmd-1* (ref. ^36^) was the exception) (Fig. 4a). The *CPEB-1* gene is specifically required for the development of oocytes and yolk glands^12^. Loss of *onecut* and *Bic-C* expression leads to a ‘no-testes’ phenotype without affecting the ovary^5,13^. The presence of a female-determining gene on a chromosome that does not recombine provides an ideal foundation for the evolution of a sex chromosome. A loss-of-function mutation in the gene on one of the two homologous chromosomes would turn the chromosome with the mutation into a male-determining chromosome. Similar logic applies to a male-determining gene.

**Extended Data Fig. 8:**
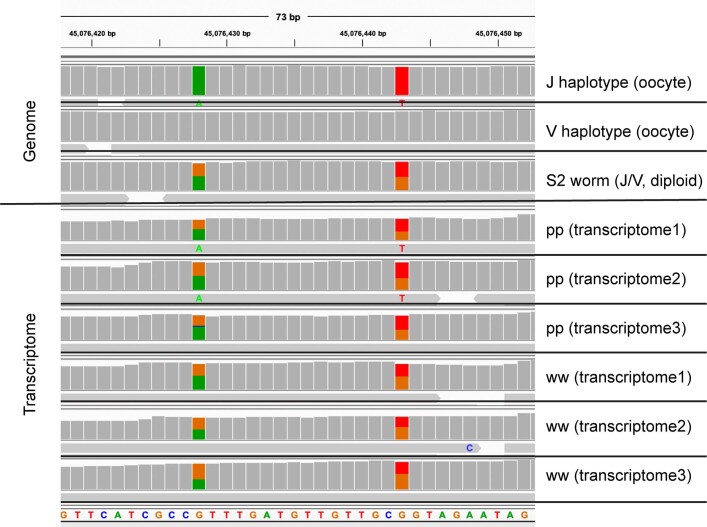
Datasets for the examination of haplotype-specific expression. The sequencing reads from genomes (J-haplotype oocytes, V-haplotype oocytes, diploid S2 worms) and transcriptomes (pp, ww from the diploid S2 worms) were aligned to Smed_chr_ref_v1. Shown in the figure are reads for the gene CPEB-2. Two variants with J and V alleles are marked by colored bars. In the genome of the adult worm S2 (i.e., DNA), both J and V alleles are present at equal ratios. In the genome of oocytes (J or V haplotype, i.e., DNA), the variants have only one allele. These observations validate the heterozygous variants. In the transcriptomes (i.e., RNA) of penis papillae (pp) and whole worms (ww) from S2 adults, both J and V alleles are present. In the three transcriptomes of pp, J alleles were more highly expressed. In the three transcriptomes of ww, the J:V allele ratios vary and do not significantly depart from equal expression.

Haplotype-specific expression of sex-specific genes is a predicted signature of a sex-primed autosome. To test this prediction, we examined the expression of 20 genes in sexually mature adult worms and in the male copulatory organ, the penis papilla^7^, in a J/V line. The 20 genes were chosen because they were characterized as having sex-specific function and/or expression in the literature and include the 8 master regulators mentioned above^5,12^ (Supplementary Table 9). Of these 20 genes, 13 contained at least one heterozygous variant in the coding sequence, which allowed us to determine whether these genes showed biased expression from the J or V haplotype (Extended Data Fig. 8). We found that a gelsolin-like protein^12^, *Smed*-*glp*, was expressed predominantly from the J haplotype in both whole worms and the penis papilla, with more haplotype-specific expression in the male copulatory organ. Four other genes showed biased expression from the J haplotype in whole worms (KY847529.1 and KY847536.1) or in the penis papilla (KY847539.1 and *CPEB-2*), and two additional genes showed biased expression from the V haplotype in the male copulatory organ (KY847532.1) or in whole worms (Smed-MSY4) (Fig. 4b and Supplementary Table 9). All seven of these genes were localized within the inversions or close to inversion breakpoints (Supplementary Table 5).

**Extended Data Fig. 9:**
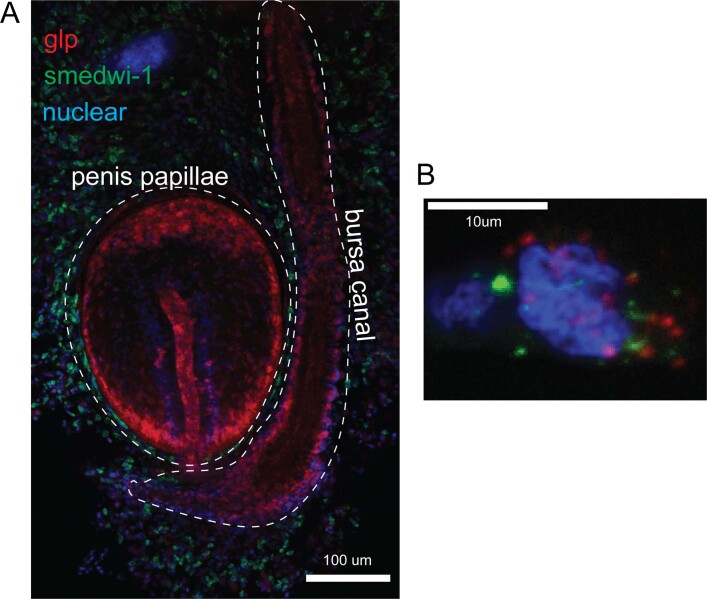
Specific expression of *smed-glp* in the male reproductive organ (penis papillae) and female reproductive organ (bursa canal). Expression of the *glp* gene (in red color) and the stem cell marker *smedwi-1* (in green color) in the dissected copulatory apparatus was visualized by Fluorescence *in situ* hybridization chain reaction. (A) White dashed lines outline penis papillae and bursa canal. Nuclei were stained by DAPI (in blue color). No *glp* expression in tissues surrounding penis papillae or bursa canal. Less than 1% of *glp+* cells express the stem cell marker *smedwi-1*. (B) Cells expressing the two markers do exist in both bursa canal and penis papillae. This experiment was repeated 3 times independently with 6 sexually mature worms, producing similar results.

We used FISH with hybridization chain reaction to confirm that the expression of *Smed-glp* is highly enriched in both the penis papilla and the bursa canal^7,37^ (Extended Data Fig. 9), which is a female organ used to receive sperm from mating partners. To examine the function of *Smed-glp*, we used RNA interference (RNAi)^14,15^ to knock down its expression in young hatchlings. After 8 weeks of feeding, both the knockdown and control hatchlings reached sexual maturity. Notably, the bursa canal was much more pronounced in the knockdown animals (Fig. 4c, d). No obvious morphological differences were observed in the male copulatory organ. These results suggest that the J allele of *Smed-glp* may have a greater role in preventing over-development of the female bursa canal (Fig. 4e), probably by controlling the number of organ-specific muscle fibres^38^. This is consistent with a previous observation that J/J individuals show higher egg production, suggesting a larger contribution to female reproduction (Fig. 4b in ref. ^24^; Supplementary Information).

We have thus identified a chromosome in the hermaphrodite planarian *S*. *mediterranea* that does not recombine, is enriched in master regulators of reproductive systems, shows allele-specific expression of sex-related genes, and contains genes orthologous to those on the sex chromosome of *Schistosoma mansoni* (Supplementary Tables 9 and 10, and Supplementary Information), which is the only known Platyhelminthes species with differentiated sex chromosomes^39^. These observations collectively led us to propose that chromosome 1 of *S*. *mediterranea* is primed for evolution into a sex chromosome.

**Extended Data Fig. 10:**
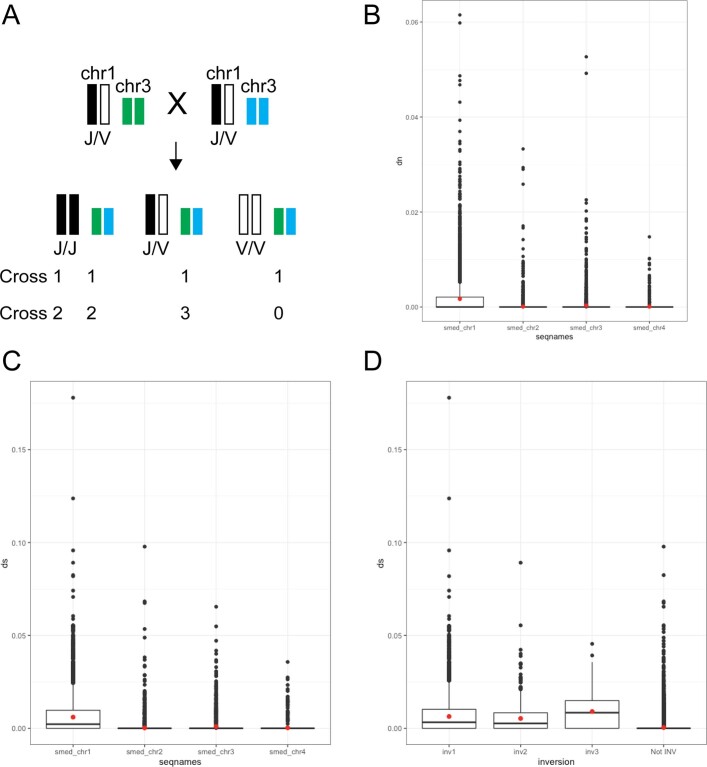
Embryonic lethality and synonymous divergence in *Schmidtea mediterranea*. (A) Two J/V lines with differential SNP markers on chromosome 3 were crossed. Zygotes were collected for genotyping. Heterozygous chromosome 3 markers validate the occurrence of fertilization. All three genotypes of chromosome 1 (J/J, J/V and V/V) were observed in zygotes. (B-C) Non-synonymous divergence (B) and Synonymous divergence (C) for all heterozygous sites in the J/V strain S2 and their distribution in different chromosomes summarized as boxplots. Chromosome 1 has the highest mean (red dots) and median dN or dS values. The min, max, median, mean, first quartile, and third quartile of dN (B) for each chromosome are as following: chr1 = (0, 0.0052, 0, 0.0017, 0, 0.0021), chr2 = (0, 0, 0, 0.000092, 0, 0), chr3 = (0, 0, 0, 0.00031, 0, 0), and chr4 = (0, 0, 0, 0.000088, 0, 0). The min, max, median, mean, first quartile, and third quartile of dS (C) for each chromosome are as following: chr1 = (0, 0.024, 0.0022, 0.006, 0, 0.0098), chr2 = (0, 0, 0, 0.00021, 0, 0), chr3 = (0, 0, 0, 0.00093, 0, 0), and chr4 = (0, 0, 0, 0.00026, 0, 0). (D) Synonymous divergence for all heterozygous sites in the J/V strain S2, and their distribution in the three chromosome 1 inversions and the rest of the genome, summarized as boxplots. Mean (red dots) and median dS decreases in the following order: inversion 3 (inv3), inversion 1 (inv1), inversion 2 (inv2), and the rest of the genome (Not INV). One way ANOVA and Turkey Honest Significant Differences tests showed that dS were significantly different between all 3 inversions (inv2-inv1: p.adj = 0.005; inv3-inv1: p.adj < 1e-06; inv3-inv2: p.adj < 1e-06). The min, max, median, mean, first quartile, and third quartile of dS for each inversion are as following: inversion 1 = (0, 0.026, 0.0034, 0.0064, 0, 0.01), inversion 2 = (0, 0.021, 0.0026, 0.0053, 0, 0.0083), inversion 3 = (0, 0.036, 0.0084, 0.0091, 0, 0.015), and non-inversion = (0, 0, 0, 0.0005, 0, 0). A total of 8317 genes from chromosome 1, 6568 genes from chromosome 2, 3394 genes from chromosome 3, and 1834 genes from chromosome 4 were plotted for non-synonymous divergence (B) and synonymous divergence (C). A total of 6633 genes from inversion 1, 754 genes from inversion 2, 298 genes from inversion 3, and 12428 genes from the rest of the genome were plotted for synonymous divergence (D).

Chromosome 1 of *S*. *mediterranea* marks an intriguing system for studying sex chromosome evolution^40^. Planarians from the islands of Sardinia and Corsica are heterozygous for the J and V haplotypes, whereas those in Sicily and Tunisia exist as J/J homozygotes. In laboratory crosses involving J/V lines, all hatchlings were J/V heterozygotes^24^. No V/V planarians have been identified either in nature or in laboratory crosses. We genotyped single zygotes from crosses between J/V individuals and determined that homozygous zygotes (J/J or V/V) exist (Extended Data Fig. 10a and Supplementary Information). We propose that early embryonic lethality leads to the loss of J/J and V/V adults in both nature and laboratory crosses. Such lethality may arise from degeneration of coding sequences on the J and V haplotypes as a consequence of crossover suppression. Indeed, chromosome 1 has an elevated rate of transposable elements and mutations introducing stop codons relative to the rest of the genome (Supplementary Table 11) as well as higher nonsynonymous substitution rates (Supplementary Table 13 and Extended Data Fig. 10b). As a consequence, the J and V haplotypes each carry unique sets of functional genes that are silenced or truncated on the other haplotype (Supplementary Table 11). 

The presence of three nested inversions on chromosome 1 suggests the possibility that recombination suppression on this chromosome may have evolved in a stepwise manner similar to that reported for the human X and Y chromosomes^17^. In support of this possibility, we observed evidence for three evolutionary strata corresponding to the inverted regions. We used PacBio genome sequencing data^26^ to identify long reads that bridged the inversion breakpoints, and we validated the three inversions identified by Hi-C (Supplementary Table 12 and Supplementary Information). We observed that the rates of heterozygous sites and synonymous substitutions were elevated within the three inversions in a pattern consistent with the evolutionary strata (Fig. 2a, Supplementary Tables 5 and 13, and Extended Data Fig. 10b, c). We used deep whole-genome sequencing data of individuals from laboratory crosses^24^ to estimate the de novo mutation rate in sexual planarians to be approximately 1.0 × 10^−8^ mutations per nucleotide per generation. On the basis of this estimate, it is likely that inversions 3, 1 and 2 evolved approximately 450,000, 320,000 and 260,000 generations ago, respectively.

Although it is not possible to know how chromosome 1 will evolve, our findings provide a snapshot of an incipient stage that supports the hypothesis that sex chromosomes evolve from homologous autosomes that acquire sex-specific roles and cease to recombine^16–19^. The locked J/V heterozygous system may facilitate this process by maintaining sex-specific alleles in the planarian population before the evolution of dioecy^41^. We propose that the planarian chromosome 1 haplotypes provide a unique opportunity to directly examine the molecular characteristics of a sex-primed autosome.

## Methods

### Planarian husbandry, RNAi and phenotyping

Sexual planarians were fed organic beef liver once a week. All animals used for experiments were selected randomly. For RNAi, hatchlings around 2 weeks old from strain S2F10 were used. RNAi food was prepared by mixing 1 µg of double-stranded RNA with 10 µl of liver paste^15^. To examine the RNAi phenotypes, photographs were taken with a stereomicroscope (Zeiss) when the animals were freely swimming. The lengths of the worms or bursa canal were measured with ImageJ software by multiple researchers in a double-blind experimental design. The ratio of the bursa canal to the whole worm was used to mitigate potential variations in worm size and their degree of relaxation caused by their soft bodies.

### Chromosome sequencing

Chromosomes were collected from multiple animals of one clonal line, S2, maintained in the laboratory by amputation and regeneration. Chromosome spreads were prepared on nuclease-free membrane slides (Zeiss) according to a previously developed protocol except that, at the last step, the tissues were dissociated into single nuclei and placed onto the slides without squashing with a coverslip^42^. Single chromosomes were identified under a ×40 lens and were collected into the caps of single PCR tubes by PALM MicroBeam laser microdissection (Zeiss). The collected chromosomes were spun down with a tabletop centrifuge in 4 µl PBS, and the DNA in the pellets was amplified with a REPLI-g Single Cell Kit (Qiagen) for sequencing on a MiSeq or HiSeq 3000 sequencing system (Illumina).

### Chromosome-scale genome assembly

A Hi-C sequencing library was prepared from multiple animals of the S2 strain using the enzyme DpnII. Sequenced reads were aligned to dd_Smes_g4.fasta^26^ with bwa mem (version 0.7.17)^43^. An assembly file was prepared from the SALSA^3^ output FINAL.fasta with juicebox_scripts (Phase Genomics). The .hic file was prepared by run-assembly-visualizer.sh from three-dimensional de novo assembly^44^. The two files were loaded into Juicebox^4^ for scaffold manipulation using split, merge, order and orient commands and for chromosome assembly. The modified assembly file (chromosome-scale) was converted to fasta (Smed_chr_ref_v1) with juicebox_assembly_converter.py.

### Hi-C library construction

A Hi-C library was generated using Phase Genomics Proximo Animal Kit version 3.0. Approximately four worms were finely chopped and were then cross-linked for 20 min at room temperature with end-over-end mixing in 1 ml of Proximo cross-linking solution. The cross-linking reaction was terminated with quenching solution for 15 min at room temperature, again with end-over-end mixing. The quenched tissue was rinsed once with 1× chromatin rinse buffer (CRB), transferred to a liquid nitrogen-cooled mortar and ground to a fine powder. The powder was resuspended in 700 µl Proximo lysis buffer 1 and lysed with glass beads for 20 min at room temperature on a vortex mixer. A low-speed spin was used to clear the large debris, and the chromatin-containing supernatant was transferred to a new tube. After a second spin at higher speed, the supernatant was removed, and the pellet containing the nuclear fraction of the lysate was washed with 1× CRB. After removal of the 1× CRB wash, the pellet was resuspended in 100 µl Proximo lysis buffer 2 and incubated at 65 °C for 15 min. Chromatin was bound to the recovery beads for 10 min at room temperature. The beads were placed on a magnetic stand and washed with 200 µl of 1× CRB.

The chromatin bound on the beads was resuspended in 150 µl Proximo fragmentation buffer, and 2.5 µl of Proximo fragmentation enzyme was added. The reaction was incubated for 1 h at 37 °C, cooled to 12 °C and then incubated with 2.5 µl of finishing enzyme for 30 min. After the addition of 6 µl of Stop Solution, the beads were washed with 1× CRB and were resuspended in 100 µl of Proximo ligation buffer supplemented with 5 µl of proximity ligation enzyme. The proximity ligation reaction was incubated at room temperature for 4 h with end-over-end mixing. To this, 5 µl of reverse cross-linking enzyme was added, and the reaction was incubated at 65 °C for 1 h.

After reversing the cross-links, the free DNA was purified with recovery beads, and the Hi-C junctions were bound to streptavidin beads and washed to remove unbound DNA. The washed beads were used to prepare paired-end deep sequencing libraries using Proximo library preparation reagents.

### Oocyte and sperm cell sequencing

Sperm cells were released from sexually mature S2 strain animals into calcium- and magnesium-free buffer (1% BSA). The cell dissociation solution was placed onto a slide and examined under a phase-contrast microscope to identify single sperm cells. Oocytes were released from egg capsules. Single sperm cells or oocytes were transferred into single PCR tubes for amplification with a REPLI-g Single Cell Kit (Qiagen). RADseq libraries and whole-genome libraries were prepared and sequenced on a HiSeq 3000 or NovaSeq S2 sequencing system (Illumina). RADseq sequencing data were analysed as described in the linkage map section. Whole-genome sequencing data were analysed as described in the recombination section.

### Synteny analysis

For candidate genes, the protein sequences of genes of interest were obtained from *Schmidtea* specimens. Such genes were aligned to *S*. *mansoni* genomes^39^ using the Protein to Nucleotide BLAST (tblastn) tool. To systematically examine the synteny of the whole genome, we used SonicParanoid (version 1.3.8)^45^ to identify one-to-one protein orthologues between *S*. *mediterranea* and *S*. *mansoni*. The *S*. *mansoni* genome assembly (V9) and its protein annotations are available at https://zenodo.org/record/5149023#.Ybk9jn3MK3I.

### Linkage map

A J/J line, D5, was crossed to a J/V line, S2F8b, to build an F_2_ population of 93 animals. Genomic DNA was extracted from a fragment of each animal using an Easy-DNA gDNA Purification Kit (K180001, ThermoFisher). Sequencing libraries for RADseq were prepared according to the procedures of Adapterama III^31^ with a few modifications. Genetic variants were identified using Stacks (version 2.41)^46,47]^. All variants were filtered with VCFtools (version 0.1.14)^48^ to remove insertions and deletions and to select biallelic SNVs. Clusters of markers located within 200 bp were removed because they were likely to correspond to repetitive elements. Markers disobeying Mendelian segregation were also removed. Only markers that were homozygous in both parents were used. The linkage map was built with R/QTL^49^.

### Quantifying recombination

Sequencing reads from S2 and its oocytes and sperm cells were aligned to Smed_chr_ref_v1 with bwa mem (version 0.7.17). Genetic variants were jointly called by using the Genome Analysis Toolkit (GATK, version 4.1.4.1) with GenomicsDB and GenotypeGVCFs^50^. Biallelic heterozygous markers in S2 were further filtered by removing abnormal markers, including those with no segregation of two alleles in the gametes, clusters of markers in close proximity (<200 bp apart) and markers of heterozygosity in sperm cells. The J and V haplotypes were manually phased for oocytes without recombination and were phased by MPR.genotyping^51^ for all gametes. The MPR.genotyping package was also used to impute or correct missing or erroneous genotypes. The final genotype bins were used to identify and visualize recombination with customized R code. Quantification of recombination was focused on crossovers between long tracks of haplotypes along a chromosome. Putative gene conversion events such as short tracks of haplotype switches encompassing <1% of the chromosome length were not included.

### Gene expression analysis

To examine the gene content on chromosome 1, transcriptome data were downloaded from the NCBI Sequence Read Archive (SRA). To examine genes related to sexual development, transcriptomes from sexual adults^9,12,52^, sexual juveniles^9^ and sexual adults with *nhr-1* RNAi^9^ were used. To examine stem cell-enriched genes, transcriptomes from sorted X1 cells and CIW4 were used^53,54^.

All sequencing data were aligned to dd_Smed_v6 (ref. ^27^) with bwa mem (version 0.7.17). Differential gene expression was analysed with DESeq2 (version 1.26.0)^55^. Expression was quantified at the transcript level with kallisto (version 0.44.0)^56^ and was imported and summarized to gene-level count matrices by tximport^57^.

### Haplotype-specific expression

To examine the haplotype-specific expression of critical regulators of the reproductive system, mRNA was extracted from six sexually mature animals of the S2 J/V line and was analysed as three biological replicates, with two animals pooled into each replicate. Nine penis papillae were dissected from nine sexual adult animals of the same line and were analysed as three biological replicates, with penis papillae from three animals pooled into each replicate. All mRNA samples were extracted on the same day and were processed at the same time for library preparation and sequencing to minimize technical variation. Libraries for RNA-seq were prepared with a Clontech SMARTer Stranded Total RNA-seq (Pico) Kit. The workflow consisted of converting total RNA to cDNA and then adding adaptors for Illumina sequencing through PCR. The PCR products were purified, and ribosomal DNA was depleted. The cDNA fragments were further amplified with primers universal to all libraries. Lastly, the PCR products were purified again to yield the final cDNA library. Different adaptors were used to multiplex samples in one lane. Sequencing was performed on an Illumina NovaSeq 6000 with a 150-bp paired-end-read run. Data quality checks were conducted using the Illumina Sequencing Analysis Viewer. Demultiplexing was performed using Illumina Bcl2fastq2 version 2.17.

All sequencing data were aligned to dd_Smed_v6 (ref. ^27^) with bwa mem (version 0.7.17). To ensure accuracy, haplotype-specific expression of the 14 genes of interest was manually examined using the Integrative Genomics Viewer. J or V allele counts were identified for each biallelic variant in the exons. For a particular gene, the allele counts were aggregated from all variants on all exons for the J and V haplotypes. The allele counts were then subjected to binomial testing and Bonferroni correction^58^ to determine whether the observed allele bias was statistically significant. *P* > 1 after Bonferroni correction was set to 1.

### De novo map, parameter optimization and phylogenetic inference

All datasets were run through the de novo pipeline as implemented in Stacks version 2.52 (refs. ^46,47^). First, paired-end reads were demultiplexed and filtered for quality using PROCESS_RADTAGS and were truncated to a length of 135 bp. Individual reads with Phred scores below 30 or uncalled bases were discarded (96.9% of reads passed the quality filters). Optimal parameters were identified following the guidelines of ref. ^59^ by running multiple iterations of the de novo pipeline and varying just one parameter with each new iteration on a subset of 13 samples from the same population, [Sic_mar], following recommendations^60^. We varied the minimum stack depth (-m) between 1 and 6 (m1–m6), the number of mismatches allowed between stacks (-M) between 1 and 10 (M1–M10), and the number of mismatches allowed to merge catalogue loci (-n) while keeping all other parameters constant (m3, M2 and n0). We then compared the number of polymorphic assembled loci across samples using a sample representation cut-off of 80% (r80) and gain or loss of polymorphic loci with each new iteration. Once -m and -M were optimized, we assessed -n by evaluating the change in the number of polymorphic loci for *n *=* M* − 1, *n* = *M* and *n* = *M* + 1. RAD loci were then assembled using the denovo_map.pl wrapper in Stacks, and the following parameters were set: *m* = 3, *M* = 2 and *n* = 3.

Assembled loci that were present in 75% of all individuals were kept from POPULATIONS, a minor allele frequency (MAF) filter of 0.04 (--min-maf) was used to filter out singleton SNPs that could mask population structure and a maximum observed heterozygosity (--max-obs-het) filter of 0.99 was used to remove potentially paralogous loci^61^. Additionally, to build the phylogenetic tree, we concatenated all RADseq loci after filtering (--phylip-var-all). Phylogenetic trees were built by maximum likelihood using RAxML-NG version 0.9.0 (ref. ^62^), starting from a random seed and applying a GTR+G substitution model and 1,000 bootstrap replicates. The sample from Menorca (Sme7-5_men) was used as an outgroup to root the tree.

### Population structure analysis

For this analysis, we excluded the outgroup sequence and ran POPULATIONS to retain loci present in all populations (-p 10) and 75% of the individuals present in each population (-r 0.75). On the basis of the loci that passed our filtering criteria, a random whitelist of 1,000 loci was generated and again run through POPULATIONS with the same criteria but retaining the first SNP at each locus (--write-single-snp). The output was exported in Structure format, and Structure version 2.3.4 (ref. ^63^) was used to infer population structure with 10,000 chains as burn-in and 100,000 MCMC chains with 20 iterations for *K* = [1–11]. The resulting files were run through Structure Harvester^64^, and the optimal *K* was determined^65^.

### DNA FISH

For telomere FISH on oocyte chromosomes, ovaries were dissected as reported previously^66,67^. Hybridization was carried out as reported with chromosome spreads^42^ except that the ovaries were kept in suspension in washing buffer or hybridization buffer. Before hybridization, the dissected ovaries were treated with a digestion buffer (0.1% SDS and 10 µg ml^−1^ proteinase K (Qiagen) in 0.3% Triton X-100 in PBS) for 10 min at room temperature. The repeat located near centromeres in Fig. 2c had the following sequence: TCTGGACGGAAATTTTTTAATCTTTATAGGCTTGTATCTCTGTCAATTTTTATTTGTTTTCATAATCTTTGATATATTTCTCGATAACTTTTGATTCTCTACATGATAGCATTTTAAAAATTGCAAAAATCATAACGGGCTCGTCAAACACAAGTCAT.

### Hybridization chain reaction and RNA FISH

To examine tissue expression of the genes *glp* and *smedwi-1*, we used probes and buffers for third-generation ISH chain reaction purchased from Molecular Instruments. Sexually mature planarians were treated with 7.5% *N*-acetyl-l-cysteine (Sigma-Aldrich) for 10 min and were then fixed in 4% paraformaldehyde (Electron Microscopy Sciences; 16% solution diluted 1:4 in PBS) for 20 min. The copulatory apparatus was dissected into a 1.5-ml RNase-free tube. The rest of the procedures followed the hybridization chain reaction RNA FISH protocol of Molecular Instruments for whole-mount mouse embryos.

### Reporting summary

Further information on research design is available in the Nature Research Reporting Summary linked to this paper.

## Online content

Any methods, additional references, Nature Research reporting summaries, source data, extended data, supplementary information, acknowledgements, peer review information; details of author contributions and competing interests; and statements of data and code availability are available at 10.1038/s41586-022-04757-3.

## Supplementary information

Supplementary Information This file contains supplementary discussion plus methods.

Reporting Summary

Supplementary Table 1 Assignment of 481 scaffolds to chromosomes with 17 sequenced chromosomes. Chromosome sample information (ploidy, identity and potential contamination), sequencing coverage and the chromosome assignment for each of the 481 scaffolds from the assembly dd_Smes_g4 are given. For chromosome assignment, 0 represents scaffolds that could not be assigned to chromosomes by ChrSeq, 5 represents scaffolds that were assigned to two chromosomes by ChrSeq and 1–4 are chromosomes.

Supplementary Table 2 Linking 481 of dd_Smes_g4 scaffolds into chromosomes with Hi-C chromatin contact sequencing. The dd_Smes_g4 scaffolds that were assigned or unassigned to Smed_chr_ref_v1 (chrAssembly), their identity in the SALSA assembly of raw Hi-C sequencing data (salsa_scaffolds) and their assignment to chromosomes by ChrSeq are given. For chromosome assignment, 0 represents scaffolds that could not be assigned to chromosomes by ChrSeq, 5 represents scaffolds that were assigned to two chromosomes by ChrSeq and 1–4 are chromosomes.

Supplementary Table 3 Summary of the final chromosome-scale genome assembly (Smed_chr_ref_v1). Hi-C detected 26 assembly errors in dd_Smes_g4, 5 of which were confirmed by ChrSeq to be inter-chromosomal misjoining. A total of 97 dd_Smes_g4 scaffolds were not assigned to Smed_chr_ref_v1.

Supplementary Table 4 Genetic markers and distances in the linkage map established from an F_2_ mapping population (supporting data for Fig. 1d). Linkage groups L.3, L.6 and L.8 contained only one genetic marker and are not included in the table. Chromosomes 2 and 4 were split into two linkage groups each, probably due to the small size of the F_2_ mapping population.

Supplementary Table 5 Distribution of genetic variants that maintained heterozygosity in the inbreeding pedigree (supporting data for Fig. 2a). The chromosomes were divided into 10-Mb windows. Heterozygous variants were identified from the inbreeding pedigree from S2 to S2F9b.

Supplementary Table 6 Chromosome 1 showing heterozygosity in all samples of an F_2_ population. The genetic markers were heterozygous in the J/V parent (parent_S2F10B_2A and parent_S2F10B_2B) and homozygous in the J/J parent (parent_D5-1), both of which are clones. Genotyping data from RADseq of 291 F_2_ samples are listed. The 291 F_2_ samples correspond to 93 unique segregants. 0/0, homozygous reference allele; 1/1, homozygous alternative allele; 0/1, heterozygous; ./., missing data.

Supplementary Table 7 Recombination in the gametes (supporting data for Fig. 2b and Extended Data Fig. 3a, b). Distributions of heterozygous variants identified in the S2 J/V line and used in the crossover assessment along chromosomes 1 and 3 in 20-Mb windows are shown. The number of crossovers identified per gamete on chromosomes 1 and 3 is also shown.

Supplementary Table 8 Chromosome 1 genotypes of wild isolates in Sardinia and Sicily (supporting data for Fig. 3d, e). Genotyping data for six different collection sites in Sardinia and two different collection sites in Sicily were aggregated. Row 1 shows the names of individual animals. 0/0, homozygous reference allele; 1/1, homozygous alternative allele; 0/1, heterozygous; ./., missing data.

Supplementary Table 9 Allele-specific expression of male and female genes (supporting data for Fig. 4a, b). Genes with well-characterized sex-related functions were identified from published work. Their locations on Smed_chr_ref_v1 and *S. mansoni* chromosomes were determined. The expressed J or V alleles for each of the nine male or female genes were quantified in the transcriptomes of whole worms and the isolated male copulatory organ, the penis papilla.

Supplementary Table 10 Distribution of orthologues between *S. mediterranea* and *S. mansoni* chromosomes. The numbers of orthologous genes shared between *S. mediterranea* and *S. mansoni* chromosomes are given. Sman_W is the sex chromosome of *S. mansoni*.

Supplementary Table 11 Distribution of transposons and stop codon mutations on different chromosomes of *S. mediterranea*. The number of repetitive elements such as transposons and short repeats on different chromosomes of g4wRepeat_chr_ref and the number of stop codons on different chromosomes of smed_chr_ref_v1 are shown. The smed_chr_ref_v1 genome was phased as J haplotype or V haplotype (Supplementary Information).

Supplementary Table 12 Bridging reads spanning the distant ends of the three inversions on chromosome 1. Publicly available PacBio sequencing data (SRX2700681–SRX2700684) were used to identify reads that span the breakpoints of the three inversions on chromosome 1. The same reads were aligned to smed_chr_ref_v1 and g4wRepeat_chr_ref. The assembly with repeats unmasked (g4wRepeat_chr_ref) identified more bridging reads, particularly for inversion 1.

Supplementary Table 13 Synonymous divergence of coding regions in the J/V strain S2 (supporting data for Extended Data Fig. 10b, c). The phased S2 genome was used to determine synonymous divergence of coding regions.

## Data Availability

The authors confirm that all data underlying the findings are fully available without restriction. All sequencing data are available from the NCBI SRA database (accession number PRJNA731187). The chromosome-scale genome assemblies for sexual *S. mediterranea* (including phased genomes and genomes with repetitive elements) are openly available on the Planosphere (https://planosphere.stowers.org/), PlanMine (https://planmine.mpibpc.mpg.de/planmine/begin.do), GenBank (GCA_022537955.1) and Zenodo (10.5281/zenodo.5807415) databases. We used publicly available NCBI PacBio sequencing data (accession numbers SRX2700681–SRX2700684) and planarian transcriptome data (accession numbers SRR2658118–SRR2658125, SRR2658134–SRR2658141, SRR3473955–SRR3473957, SRR3629945–SRR3629952, SRR6351185–SRR6351188, SRR6351201–SRR6351204, SRR6351213–SRR6351216, SRR6363910–SRR6363927 and SRR6364586–SRR6364588).

## Code availability

Custom code for data analysis can be openly accessed on the Zenodo database at 10.5281/zenodo.5807415.

## References

[1] Lazaro EM (2011). *Schmidtea mediterranea* phylogeography: an old species surviving on a few Mediterranean islands?. BMC Evol. Biol..

[2] Benazzi M, Baguná J, Ballester R, Puccinelli I, Papa RD (1975). Further contribution to the taxonomy of the «*Dugesia Lugubris*-*Polychroa Group*» with description of *Dugesia Mediterranea* N.S.P. (Tricladida, Paludicola). Boll. Zool..

[3] Burton JN (2013). Chromosome-scale scaffolding of de novo genome assemblies based on chromatin interactions. Nat. Biotechnol..

[4] Durand NC (2016). Juicebox provides a visualization system for Hi-C contact maps with unlimited zoom. Cell Syst..

[5] Wang Y, Zayas RM, Guo T, Newmark PA (2007). *Nanos* function is essential for development and regeneration of planarian germ cells. Proc. Natl Acad. Sci. USA.

[6] Collins JJ (2010). Genome-wide analyses reveal a role for peptide hormones in planarian germline development. PLoS Biol..

[7] Chong T, Stary JM, Wang Y, Newmark PA (2011). Molecular markers to characterize the hermaphroditic reproductive system of the planarian *Schmidtea mediterranea*. BMC Dev. Biol..

[8] Tharp ME, Collins JJ, Newmark PA (2014). A lophotrochozoan-specific nuclear hormone receptor is required for reproductive system development in the planarian. Dev. Biol..

[9] Zhang, S. et al. A nuclear hormone receptor and lipid metabolism axis are required for the maintenance and regeneration of reproductive organs. Preprint at *bioRxiv*10.1101/279364 (2018).

[10] Iyer H, Issigonis M, Sharma PP, Extavour CG, Newmark PA (2016). A premeiotic function for boule in the planarian *Schmidtea mediterranea*. Proc. Natl Acad. Sci. USA.

[11] Saberi A, Jamal A, Beets I, Schoofs L, Newmark PA (2016). GPCRs direct germline development and somatic gonad function in planarians. PLoS Biol..

[12] Rouhana L, Tasaki J, Saberi A, Newmark PA (2017). Genetic dissection of the planarian reproductive system through characterization of *Schmidtea mediterranea* CPEB homologs. Dev. Biol..

[13] Li P (2021). Single-cell analysis of *Schistosoma mansoni* identifies a conserved genetic program controlling germline stem cell fate. Nat. Commun..

[14] Newmark PA, Reddien PW, Cebrià F, Alvarado AS (2003). Ingestion of bacterially expressed double-stranded RNA inhibits gene expression in planarians. Proc. Natl Acad. Sci. USA.

[15] Rouhana L (2013). RNA interference by feeding in vitro-synthesized double-stranded RNA to planarians: methodology and dynamics. Dev. Dynam..

[16] Bachtrog D (2006). A dynamic view of sex chromosome evolution. Curr. Opin. Genet. Dev..

[17] Lahn BT, Page DC (1999). Four evolutionary strata on the human X chromosome. Science.

[18] Rice WR (1996). Evolution of the Y sex chromosome in animals. BioScience.

[19] Charlesworth B (1991). The evolution of sex chromosomes. Science.

[20] Muller HJ (1918). Genetic variability, twin hybrids and constant hybrids, in a case of balanced lethal factors. Genetics.

[21] Charlesworth D (2017). Evolution of recombination rates between sex chromosomes. Philos. Trans. R. Soc. Lond. B.

[22] Reddien PW, Sanchez Alvarado A (2004). Fundamentals of planarian regeneration. Annu. Rev. Cell Dev. Biol..

[23] Newmark PA, Sanchez Alvarado A (2002). Not your father’s planarian: a classic model enters the era of functional genomics. Nat. Rev. Genet..

[24] Guo L, Zhang S, Rubinstein B, Ross E, Alvarado AS (2016). Widespread maintenance of genome heterozygosity in *Schmidtea mediterranea*. Nat. Ecol. Evol..

[25] Zayas RM (2005). The planarian *Schmidtea mediterranea* as a model for epigenetic germ cell specification: analysis of ESTs from the hermaphroditic strain. Proc. Natl Acad. Sci. USA.

[26] Grohme MA (2018). The genome of *Schmidtea mediterranea* and the evolution of core cellular mechanisms. Nature.

[27] Brandl H (2016). PlanMine—a mineable resource of planarian biology and biodiversity. Nucleic Acids Res..

[28] Robb SM, Ross E, Sanchez Alvarado A (2008). SmedGD: the *Schmidtea mediterranea* genome database. Nucleic Acids Res..

[29] Weise A (2010). High-throughput sequencing of microdissected chromosomal regions. Eur. J. Hum. Genet..

[30] Ma L (2010). Direct determination of molecular haplotypes by chromosome microdissection. Nat. Methods.

[31] Bayona-Vasquez NJ (2019). Adapterama III: quadruple-indexed, double/triple-enzyme RADseq libraries (2RAD/3RAD). PeerJ.

[32] Dobzhansky T, Epling C (1948). The suppression of crossing over in inversion heterozygotes of *Drosophila pseudoobscura*. Proc. Natl Acad. Sci. USA.

[33] Miller DE (2018). The molecular and genetic characterization of second chromosome balancers in *Drosophila melanogaster*. G3.

[34] Sun Y, Svedberg J, Hiltunen M, Corcoran P, Johannesson H (2017). Large-scale suppression of recombination predates genomic rearrangements in *Neurospora tetrasperma*. Nat. Commun..

[35] Zheng X (2012). A high-performance computing toolset for relatedness and principal component analysis of SNP data. Bioinformatics.

[36] Chong T, Collins JJ, Brubacher JL, Zarkower D, Newmark PA (2013). A sex-specific transcription factor controls male identity in a simultaneous hermaphrodite. Nat. Commun..

[37] Hyman, L. H. in *The Invertebrates,* vol. II (ed. Boell, E. J.) 52–458 (McGraw-Hill Book Company, 1951).

[38] Bertin B (2021). *Gelsolin* and *dCryAB* act downstream of muscle identity genes and contribute to preventing muscle splitting and branching in *Drosophila*. Sci. Rep..

[39] Buddenborg, S. et al. Assembled chromosomes of the blood fluke *Schistosoma mansoni* provide insight into the evolution of its ZW sex-determination system. Preprint at *bioRxiv*10.1101/2021.08.13.456314 (2021).

[40] Charlesworth B, Charlesworth D (1978). A model for the evolution of dioecy and gynodioecy. Am. Nat..

[41] Charlesworth, D. & David, S. in *Sex Determination in Plants*, 1st ed. (ed. Ainsworth, C. C.) 25–50 (Garland Science, 1999).

[42] Guo L (2018). An adaptable chromosome preparation methodology for use in invertebrate research organisms. BMC Biol..

[43] Li H, Durbin R (2009). Fast and accurate short read alignment with Burrows–Wheeler transform. Bioinformatics.

[44] Dudchenko O (2017). De novo assembly of the *Aedes aegypti* genome using Hi-C yields chromosome-length scaffolds. Science.

[45] Cosentino S, Iwasaki W (2019). SonicParanoid: fast, accurate and easy orthology inference. Bioinformatics.

[46] Catchen J, Hohenlohe PA, Bassham S, Amores A, Cresko WA (2013). Stacks: an analysis tool set for population genomics. Mol. Ecol..

[47] Catchen JM, Amores A, Hohenlohe P, Cresko W, Postlethwait JH (2011). Stacks: building and genotyping loci de novo from short-read sequences. G3.

[48] Danecek P (2011). The variant call format and VCF tools. Bioinformatics.

[49] Broman KW, Wu H, Sen S, Churchill GA (2003). R/qtl: QTL mapping in experimental crosses. Bioinformatics.

[50] McKenna A (2010). The genome analysis toolkit: a MapReduce framework for analyzing next-generation DNA sequencing data. Genome Res..

[51] Xie W (2010). Parent-independent genotyping for constructing an ultrahigh-density linkage map based on population sequencing. Proc. Natl Acad. Sci. USA.

[52] Davies EL (2017). Embryonic origin of adult stem cells required for tissue homeostasis and regeneration. eLife.

[53] Zeng A (2018). Prospectively isolated tetraspanin^+^ neoblasts are adult pluripotent stem cells underlying planaria regeneration. Cell.

[54] Duncan EM, Chitsazan AD, Seidel CW, Sanchez Alvarado A (2015). Set1 and MLL1/2 target distinct sets of functionally different genomic loci in vivo. Cell Rep..

[55] Love MI, Huber W, Anders S (2014). Moderated estimation of fold change and dispersion for RNA-seq data with DESeq2. Genome Biol..

[56] Bray NL, Pimentel H, Melsted P, Pachter L (2016). Near-optimal probabilistic RNA-seq quantification. Nat. Biotechnol..

[57] Soneson C, Love MI, Robinson MD (2015). Differential analyses for RNA-seq: transcript-level estimates improve gene-level inferences. F1000Res.

[58] Etymologia: Bonferroni correction. *Emerg. Infect. Dis*. **21**, 289 (2015). 10.3201/eid2102.ET2102 PMC4313667 25786274

[59] Paris JR, Stevens JR, Catchen JM (2017). Lost in parameter space: a road map for STACKS. Methods Ecol. Evol..

[60] Rochette NC, Catchen JM (2017). Deriving genotypes from RAD-seq short-read data using Stacks. Nat. Protoc..

[61] Stobie CS, Oosthuizen CJ, Cunningham MJ, Bloomer P (2018). Exploring the phylogeography of a hexaploid freshwater fish by RAD sequencing. Ecol. Evol..

[62] Kozlov AM, Darriba D, Flouri T, Morel B, Stamatakis A (2019). RAxML-NG: a fast, scalable and user-friendly tool for maximum likelihood phylogenetic inference. Bioinformatics.

[63] Pritchard JK, Stephens M, Donnelly P (2000). Inference of population structure using multilocus genotype data. Genetics.

[64] Earl DA, vonHoldt BM (2012). Structure Harvester: a website and program for visualizing Structure output and implementing the Evanno method. Conserv. Genet. Resour..

[65] Evanno G, Regnaut S, Goudet J (2005). Detecting the number of clusters of individuals using the software Structure: a simulation study. Mol. Ecol..

[66] Guo, L. et al. Subcellular analyses of planarian meiosis implicates a novel, double-membraned vesiculation process in nuclear envelope breakdown. Preprint at *bioRxiv*10.1101/620609 (2019).

[67] Guo F (2021). Planarian ovary dissection for ultrastructural analysis and antibody staining. J. Vis. Exp..

[68] Ouellette LA, Reid RW, Blanchard SG, Brouwer CR (2018). LinkageMapView—rendering high-resolution linkage and QTL maps. Bioinformatics.

