# Supplementary material for: Island-specific evolution of a sex-primed autosome in a sexual planarian
Source: Nature. 2022 Jun 1;606(7913):329–34. doi: 10.1038/s41586-022-04757-3 (PMC9177419; doi:10.1038/s41586-022-04757-3)
Supplement: Supplementary file 1 — This file contains supplementary discussion plus methods. [file 41586_2022_4757_MOESM1_ESM.pdf]

nature portfolio

https://doi.org/10.1038/s41586-022-04757-3

Supplementary information

Island-specific evolution of a sex-primed autosome in a sexual planarian

In the format provided by the authors and unedited

JJ individuals make a greater contribution to female reproduction than JV individuals

Both JJ and JV individuals are simultaneous hermaphrodites in anatomy and in reproduction. To examine if JJ individuals have biases toward male or female productivity or if J and V haplotypes have diverged to be asymmetrical in JV individuals, we used fertility data from JJ x JJ, JJ x JV and JV x JV crosses. These data were presented in Figure 4b, Guo et. al. 2017[1]. We collected fertility data by quantifying the total number of eggs produced by each mating pair, average number of hatchlings per egg capsule, and the percentage of hatched egg capsules.

In the mating experiments between clones, crosses between JJ isolates (i.e., D5D, D5I) were sterile (Figure 4b, middle column), but crosses between JV isolates (i.e., S2, S2F8a, D2E) were fertile (Figure 4b, left column). JJ isolates produced significantly more eggs than 3 different JV isolates. These data suggest that JJ isolates are biased toward egg production relative to JV isolates.

Crosses between JJ isolates (i.e., D5D, D5I) and JV isolates (i.e., S2F8a) showed that when JJ isolates serve as mothers, the number of hatchlings per egg capsule and the percentage of hatched eggs are considerably less than with JV isolates as mothers. However, the total number of eggs produced by JJ isolates in such crosses is significantly greater than with JV isolates as mothers (Figure 4b, right column). These data provide further support that JJ isolates are biased toward female productivity (e.g., egg production). When the two JJ isolates, D5D and D5I, are compared to a different JV isolate, D2E, in crosses to the JV isolate S2F8a, this bias of JJ individuals toward egg production is prominent. These observations are consistent with the results in the left and middle columns of Figure 4b from crosses of clones.

The mating experiments between different genotypes were carried out as follows: two animals were paired for mating 24 hours after feeding with colored food. One animal was colored blue while the other animal was colored red. After 24 hours of mating, the two individuals were separated into different dishes for egg laying for 48 hours, before their next feeding. The animals can retain food color for 5 days, allowing us to track genotypes and eggs produced by each genotype.

To summarize, the data described in Figure 4b[1] suggest that JJ isolates or J haplotypes are biased toward female functions (e.g., egg production). As Guo et. al. 2017 was the first paper on the JV genetic phenomenon, it focused on the JV heterozygosity; this earlier paper did not consider fertility differences between JJ and JV individuals that are presented here based on re-examination of the data in that paper.

Phasing the planarian genome

All currently available planarian genome references were built with sequencing reads from either S2F2 or S2F17 lines, which are inbred progeny of the line S2. To establish a phased genome reference, we took advantage of homozygous chromosomes in multiple planarian lines. First, we used alleles from the D5 lines (i.e., D5D, D5I)[1] for chromosome 1 phasing, as >90% of the sites heterozygous in S2 or S2-derived lines are homozygous in D5 lines on chromosome 1. D5 haplotypes are shared by one haplotype of the S2 oocytes and are complementary to the other

haplotype of the S2 oocytes. Second, we used alleles from S2F9a lines[1] for phasing chromosomes 2, 3, and 4, as S2F9a is a highly inbred line from line S2. Lastly, the homozygous haplotypes of D5 chromosome 1 and homozygous haplotypes of S2F9a chromosomes 2-4 were combined to produce a phased reference, with all short insertions and deletions removed from the variant calls. That is, the phased genome reference only considered variations present as single nucleotide polymorphisms. The complementary phase of the genome reference was created by using the alternative alleles of the shared heterozygous sites of line S2. These two versions of phased genome references are created from smed_chr_ref_v1.fa with s2.Jhaplotype.recode.vcf and s2.Vhaplotype.recode.vcf, by Genome Analysis Tool Kit (GATK, v3.4.1)[2]. The D5 chromosome 1 alleles are J haplotypes[1]. These two phased references allow for reliable analysis of synonymous divergence in coding genes on different chromosomes of S2. These two genomes are uploaded to Zenodo (10.5281/zenodo.5807415).

Synonymous divergence analysis
To understand the relationship between the inversions on chromosome 1 and neutral diversity, we calculated the synonymous divergence between the J and V haplotypes. In more detail, we extracted primary transcripts from the phased J and V genomes using gffread (v0.12.7)[3]. This resulted in a list of gene sequences, one for each haplotype. We calculated the synonymous divergence between the J and V genes using the codonseq package of Biopython[4] (v1.7.9) with the NG86 method[5].

Transposon analysis
A chromosome scale genome assembly with unmasked scaffolds was established with procedures similar to those used to produce the assembly with masked repeats (i.e, smed_chr_ref_v1). From here on, this new unmasked genome assembly is designated “g4wRepeats_chr_ref”. We used RepeatMasker (v4.1.2-p1) and the transposon database D5fam.h5 (release 3.5, Oct 2021) to examine transposable elements in the planarian genome. The search engine used was HMMER v3.3.2. The unmasked chromosome scale genome assembly (g4wRepeats_chr_ref) is now deposited in NCBI (PRJNA731187) and Zenodo (10.5281/zenodo.5807415).

The existence of JJ zygotes and VV zygotes in JV x JV crosses
Two J/V lines (S2F9b and S2F10a) were crossed to produce eggs. Zygotes were collected for whole genome DNA amplification. Libraries were prepared with a RAD-seq protocol[6]. As zygotes collected from egg capsules can be unfertilized oocytes, we used SNP markers on chromosome 3 to determine the occurrence of fertilization between gametes from the two parents. Among such verified zygotes, we determined that all three genotypes of J and V haplotypes exist: J/J, J/V and V/V.

Validation of inversions by bridging reads from PACBio long reads
The inversion breakpoints on chromosome 1 in the Smed_chr_ref_v1 assembly fall in the following regions: inversion 1 (13,375,000-13,400,000 & 319,350,000-319,375,000); inversion 2 (26,850,000-26,900,000 & 56,350,000-56,400,000); inversion 3 (243,875,000-243,900,000 & 254,100,000-254,125,000).

To validate the inversions, we used long reads from PAC Bio sequencing that are publicly available for line S2F17, an inbred descendant of line S2, which maintains the J/V haplotypes. The accession numbers are SRX2700681-SRX2700684. The sequencing reads were aligned to Smed_chr_ref_v1 with minimap2(v2.23)[7]. Custom code was used to screen the aligned bam file for reads that bridge the two distant ends of an inversion. This led to the identification of 2 reads validating inversion 3 and 10 reads validating inversion 2. The 12 bridging reads are from 12 independent sequencing runs.

We hypothesized that some reads spanning inversion breakpoints were missed due to repeat masking; breakpoints are expected to fall within repetitive sequence arrays and/or transposable elements. To improve the identification of bridging reads, especially for inversion 1, we used the newly assembled unmasked assembly, g4wRepeats_chr_ref. We aligned the same set of PAC Bio reads to g4wRepeats_chr_ref and ran the custom code to screen for bridging reads. We uncovered 169 bridging reads for inversion 1, 1 bridging read for inversion 2, and 6 bridging reads for inversion 3.

Enrichment of chromosome 1 orthologous genes on the Z chromosome of *S. mansoni*

The proportion of orthologs of *S. mansoni* Z chromosome genes that are located on *S. mediterranea* (Smed) chromosome 1 is greater than the overall proportion of all Smed genes that are located on this chromosome (40% vs. 36.8%, fold-enrichment = 1.09, Supplementary Table 10, “wholeGenome”). We repeated this analysis for subsets of genes related to sexual reproduction. We first identified 3 sets of sex-related genes by differential gene expression analysis of three independent transcriptome datasets from Smed. This analysis is unbiased with respect to chromosomal locations of the genes. The first experiment compared adult sexual planarians with those treated with nhr1 RNAi, which abolishes all reproductive systems (Zhang et. al. 2018, ref 31). The second experiment compared adult sexual planarians with juvenile planarians, which have not developed sexual reproductive systems (Zhang et. al. 2018, ref 31; Davies et. al. 2017, ref 57; Rouhana et. al. 2017, ref 35). The third experiment, performed by us for this study, compared expression in whole adult sexual planarians with expression in the penis papilla, a male sexual organ. For all three sets of sex-related genes, the genes shared with the *S. mansoni* Z chromosome were substantially enriched on the Smed chromosome 1, to a greater extent than in the analysis of all Smed genes (fold-enrichment = 1.78, 1.34, and 1.17 respectively). See Supplementary Table 10 and the methods section “Synteny analysis”. Although the observed enrichments did not reach statistical significance due to the small total number of orthologous genes, the trend toward enrichment is consistent across the different gene sets. These observations are consistent with our proposal that chromosome 1 is a sex-primed autosome, rather than a fully differentiated sex chromosome.

Reference

1 Guo, L., Zhang, S., Rubinstein, B., Ross, E. & Alvarado, A. S. Widespread maintenance of genome heterozygosity in Schmidtea mediterranea. *Nat Ecol Evol* **1**, 19, doi:10.1038/s41559-016-0019 (2016).

2 GA, V. d. A. & BD, O. C. *Genomics in the Cloud: Using Docker, GATK, and WDL in Terra*. first edn, (O'Reilly Media., 2020).
3 Pertea, G. & Pertea, M. GFF Utilities: GffRead and GffCompare. *F1000Res* **9**, doi:10.12688/f1000research.23297.2 (2020).
4 Cock, P. J. *et al.* Biopython: freely available Python tools for computational molecular biology and bioinformatics. *Bioinformatics* **25**, 1422-1423, doi:10.1093/bioinformatics/btp163 (2009).
5 Nei, M. & Gojobori, T. Simple methods for estimating the numbers of synonymous and nonsynonymous nucleotide substitutions. *Mol Biol Evol* **3**, 418-426, doi:10.1093/oxfordjournals.molbev.a040410 (1986).
6 Bayona-Vasquez, N. J. *et al.* Adapterama III: Quadruple-indexed, double/triple-enzyme RADseq libraries (2RAD/3RAD). *PeerJ* **7**, e7724, doi:10.7717/peerj.7724 (2019).
7 Li, H. Minimap2: pairwise alignment for nucleotide sequences. *Bioinformatics* **34**, 3094-3100, doi:10.1093/bioinformatics/bty191 (2018).
