# Supplementary material for: Island-specific evolution of a sex-primed autosome in a sexual planarian
Source: Nature. 2022 Jun 1;606(7913):329–34. doi: 10.1038/s41586-022-04757-3 (PMC9177419; doi:10.1038/s41586-022-04757-3)
Supplement: Supplementary file 2 — Reporting Summary [file 41586_2022_4757_MOESM2_ESM.pdf]

nature portfolio

Corresponding author(s): Leonid Kruglyak, Longhua Guo

Last updated by author(s): 3/11/2022

Reporting Summary

Nature Portfolio wishes to improve the reproducibility of the work that we publish. This form provides structure for consistency and transparency in reporting. For further information on Nature Portfolio policies, see our Editorial Policies and the Editorial Policy Checklist.

Statistics

For all statistical analyses, confirm that the following items are present in the figure legend, table legend, main text, or Methods section.

n/a	Confirmed	
☐	☒	The exact sample size (*n*) for each experimental group/condition, given as a discrete number and unit of measurement
☐	☒	A statement on whether measurements were taken from distinct samples or whether the same sample was measured repeatedly
☐	☒	The statistical test(s) used AND whether they are one- or two-sided<br>*Only common tests should be described solely by name; describe more complex techniques in the Methods section.*
☒	☐	A description of all covariates tested
☐	☒	A description of any assumptions or corrections, such as tests of normality and adjustment for multiple comparisons
☐	☒	A full description of the statistical parameters including central tendency (e.g. means) or other basic estimates (e.g. regression coefficient) AND variation (e.g. standard deviation) or associated estimates of uncertainty (e.g. confidence intervals)
☐	☒	For null hypothesis testing, the test statistic (e.g. *F*, *t*, *r*) with confidence intervals, effect sizes, degrees of freedom and *P* value noted<br>*Give P values as exact values whenever suitable.*
☒	☐	For Bayesian analysis, information on the choice of priors and Markov chain Monte Carlo settings
☒	☐	For hierarchical and complex designs, identification of the appropriate level for tests and full reporting of outcomes
☒	☐	Estimates of effect sizes (e.g. Cohen's *d*, Pearson's *r*), indicating how they were calculated

Our web collection on statistics for biologists contains articles on many of the points above.

Software and code

Policy information about availability of computer code

Data collection	Image J (Fiji, 1.53f51)
Data analysis	bwa mem (version 0.7.17), SALSA, Juicebox, 3d-dna, sonic paranoid (v1.3.8), Stacks (version 2.41; 2.52), VCFtools (version 0.1.14), GATK (v.4.1.4.1), DEseq2 (version 1.26.0), kallisto (version 0.44.0), Bcl2fastq2 (version 2.17), RAxML-NG (version 0.9.0), STRUCTURE (version 2.3.4), custom codes (https://zenodo.org/record/5807415#.Yig0VRDMK3I)

For manuscripts utilizing custom algorithms or software that are central to the research but not yet described in published literature, software must be made available to editors and reviewers. We strongly encourage code deposition in a community repository (e.g. GitHub). See the Nature Portfolio guidelines for submitting code & software for further information.

Data

Policy information about availability of data

All manuscripts must include a data availability statement. This statement should provide the following information, where applicable:

- Accession codes, unique identifiers, or web links for publicly available datasets
- A description of any restrictions on data availability
- For clinical datasets or third party data, please ensure that the statement adheres to our policy

The authors confirm that all data underlying the findings are fully available without restriction. All sequencing data are available from the NCBI SRA database (accession number PRJNA731187). The chromosome-scale genome assemblies for sexual Schmidtea mediterranea (including phased genomes and genomes with repetitive elements) are openly available at: planosphere(planosphere.stowers.org); planmine (planmine.mpibpc.mpg.de/planmine/begin.do); Zenodo (10.5281/zenodo.5807415). Publicly available PacBio sequencing data from NCBI were used (accessions: SRX2700681-SRX2700684). Publicly available planarian transcriptome data from NCBI were used (accessions: SRR2658118-SRR2658125, SRR2658134-SRR2658141, SRR3473955-SRR3473957, SRR3629945-SRR3629952,

SRR6351185-SRR6351188, SRR6351201-SRR6351204, SRR6351213-SRR6351216, SRR6363910-SRR6363927, SRR6364586-SRR6364588).

Field-specific reporting

Please select the one below that is the best fit for your research. If you are not sure, read the appropriate sections before making your selection.

☒ Life sciences ☐ Behavioural & social sciences ☐ Ecological, evolutionary & environmental sciences

For a reference copy of the document with all sections, see nature.com/documents/nr-reporting-summary-flat.pdf

Life sciences study design

All studies must disclose on these points even when the disclosure is negative.

Sample size	The numbers of chromosomes, sperm, oocytes, wild isolates and animals used in each experiment range from 3 to more than 20. The numbers were chosen to ensure representative samples; the specific choices for each experiment were guided by practical considerations of reproducibility and feasibility.
Data exclusions	Failed sequencing samples were excluded if the DNA amplification failed or the sequencing resulted with little data. No data exclusions were performed on successful samples.
Replication	All experiments were repeated at least three times. All attempts to replicate the experiments were successful.
Randomization	All chromosomes, sperm, oocytes and animals were selected randomly. For experimental and control groups, animals were allocated randomly.
Blinding	The measurements of the length of female bursa canal or worms were double-blinded and repeated at least twice. In other experiments, blinding is not essential. For example, in the analysis of recombination or wild diversity, all gametes, animals, chromosomes were processed together as one dataset. The researchers were essentially blinded to the relevance of a particular variant, chromosome or animal.

Reporting for specific materials, systems and methods

We require information from authors about some types of materials, experimental systems and methods used in many studies. Here, indicate whether each material, system or method listed is relevant to your study. If you are not sure if a list item applies to your research, read the appropriate section before selecting a response.

Materials & experimental systems

n/a	Involved in the study	
☒	☐	Antibodies
☒	☐	Eukaryotic cell lines
☒	☐	Palaeontology and archaeology
☐	☒	Animals and other organisms
☒	☐	Human research participants
☒	☐	Clinical data
☒	☐	Dual use research of concern

Methods

n/a	Involved in the study	
☒	☐	ChIP-seq
☒	☐	Flow cytometry
☒	☐	MRI-based neuroimaging

Animals and other organisms

Policy information about studies involving animals; ARRIVE guidelines recommended for reporting animal research

Laboratory animals	The animals used in the study are the sexual biotype of Schmidtea mediterranea, which are simultaneous hermaphrodites. Sexually mature adults (4 month old to 1 year old) were used.
Wild animals	No live wild animals were used. DNA samples previously extracted from wild animals were sequenced and analyzed.
Field-collected samples	Wild isolates of Schmidtea mediterranea were collected from the field and immediately fixed in 70% ethanol. DNA were extracted from these animals in earlier studies. Such DNA samples were used in the current study.
Ethics oversight	As Schmidtea mediterranea is a free-living invertebrate and harmless to humans/environments, no university ethical approval is needed. We were mindful to give the animals best housing conditions (e.g., constant optimal temperature, clean water, consistent feeding schedules, ample space) and best euthanasia practice (e.g., quick treatment with 10% bleach). We followed guidelines of ARRIVE in our reporting.

Note that full information on the approval of the study protocol must also be provided in the manuscript.
